# Evaluating Pro- and Re-Active Driving Behavior by Means of the EEG

**DOI:** 10.3389/fnhum.2018.00205

**Published:** 2018-05-24

**Authors:** Edmund Wascher, Stefan Arnau, Ingmar Gutberlet, Melanie Karthaus, Stephan Getzmann

**Affiliations:** ^1^IfADo—Leibniz Research Centre for Working Environment and Human Factors, Dortmund, Germany; ^2^BlindSight GmbH, Schlitz, Germany

**Keywords:** car driving, mental fatigue, cognitive controllability, EEG, alpha and theta power

## Abstract

Traffic safety essentially depends on the drivers’ alertness and vigilance, especially in monotonous or demanding driving situations. Brain oscillatory EEG activity offers insight into a drivers’ mental state and has therefore attracted much attention in the past. However, EEG measures do not only vary with internal factors like attentional engagement and vigilance but might also interact with external factors like time on task, task demands, or the degree to which a traffic situation is predictable. In order to identify EEG parameters for cognitive mechanisms involved in tasks of high and low controllability, the present study investigated the interaction of time on task, task load, and cognitive controllability in simulated driving scenarios, using an either re-active or pro-active driving task. Participants performed a lane-keeping task, half of them compensating varying levels of crosswind (re-active task), and the other half driving along a winding road (pro-active task). Both driving tasks were adjusted with respect to difficulty. The analysis of oscillatory EEG parameters showed an increase in total power (1–30 Hz) with time on task, with decreasing task load, and in the re-active compared to the pro-active task. Furthermore, the relative power in Alpha band increased with decreasing task load and time on task, while relative Theta power showed the opposite pattern. Moreover, relative Alpha power was also higher in the re-active, than pro-active, driving situation, an effect that even increased with time on task. The results demonstrate that the controllability of a driving situation has a similar effect on oscillatory EEG activity like time on task and task load.

## Introduction

Sleepiness and fatigue have been identified as the reason behind 10%–40% of fatal crashes and highway accidents caused by car or truck drivers (Horne and Reyner, 1999; Philip, 2005). Sleep related factors, circadian rhythms and driving duration may play a role for drivers’ fatigue as well as boredom and monotony when long term rides are considered (Otmani et al., 2005; Philip, 2005; Papadelis et al., 2007). May and Baldwin (2009) proposed that both active and passive factors can generate a cognitive decline with time on task. Not only longer lasting cognitively demanding activity may lead to more error prone behavior, but also monotonous surroundings may evoke hypo-vigilance (Larue et al., 2011, a state that may be phenomenologically comparable to the former one. Thus, vigilance and attentional engagement vary not only with time on task but also with the driving situation (Larue et al., 2011; Wascher et al., 2016).

One core factor that determines attentional engagement is the degree to which a situation is under control of the actor. Human behavior can be driven by two sources in a general sense: an action can be: (a) the answer to an outer stimulation as a “re-action”, or (b) generated by an inner source pro-actively as an intentional act (Braver, 2012; Garcia et al., 2017). In real life, no action ever will be exclusively either re-active or pro-active. The weighting of these two elements reflects controllability of a situation, in particular when a task is complex (Garcia et al., 2017). A proactive allocation of attentional resources may boost performance but is costly. The likelihood of its deployment therefore depends on the size of the expected increase of the outcome compared to reactive action-control, as well as on the validity of the stimulus indicating the need for control (Braver, 2012). When you are driving a car, the same steering action might be required when you take a curve or when you realize that your car is displaced by the sudden onset of crosswind. While the former action can be planned in advance, responding to crosswind is highly determined by the unintended motion path of the car and therefore purely re-active. However, also without crosswind, turn driving can be more or less re-active when a bend changes its radius unexpectedly or when the velocity is not adapted to visibility (e.g., with fog).

In general, the pro-active allocation of cognitive control is directed forwards. It intentionally adapts to an upcoming event (Garcia et al., 2017). In experimental studies, it has been shown that stimulus processing is accelerated and response selection requirements are markedly reduced (Waszak et al., 2005) primarily due to preparatory activity, initiated by the posterior medial frontal cortex (Oliveira et al., 2014). The ability to prepare for an action, however, is not only a matter of the situation but also strongly determined by individual resources. In a driving task, advances of action planning were demonstrated comparing poor and good navigators (Ou et al., 2012). Inefficient preparation of poor navigators led to increased need to re-act in a decision situation. Cognitive control resources that are needed for such decisions are prone to be reduced by external factors such as mental fatigue, stress, or increasing age. For example, when vigilance is lowered due to long term or monotonous driving, cognitive control has been reported to be substantially lowered (Boksem et al., 2005; Bonnefond et al., 2011). Human intentional control in a given situation thus highly depends on the demands of a task and the specific resources available (Garcia et al., 2017).

Psychophysiological studies that intend to estimate the drivers’ state by means of the EEG are well established (Borghini et al., 2014; Ahn et al., 2016). They promise an objective measure of e.g., drivers’ fatigue and have even been proposed as a potential countermeasure promoting accident avoidance (Lal and Craig, 2001). Different measures like power, properties of Alpha spindles (Schmidt et al., 2009; Simon et al., 2011) or connectivity in various frequency bands (Kong et al., 2015), phase locking among others in the gamma band (Kong et al., 2012) or combinations of these parameters have been proposed as indicators of a driver’s mental state. In order to develop online tracking systems, temporally highly resolving analyses have been provided (Liang et al., 2005; Lin et al., 2005; Papadelis et al., 2007; AlZu’bi et al., 2014). Most recently, the availability of cheap and simple to be applied EEG systems might constitute an opportunity to transfer these methods to real life scenarios (Picot et al., 2013; AlZu’bi et al., 2014; Nugraha et al., 2016).

Some of the measures mentioned above, however, additionally vary with cognitive demands. Alpha power decreases with the allocation of attention (Herrmann and Knight, 2001) and with increasing working memory demands (Klimesch, 2012). This leads to the assumption that high Alpha power may be related to attentional withdrawal or task disengagement (Wascher et al., 2014, 2016) that becomes predominant when boring tasks have to be performed (Borghini et al., 2014). In other words, Alpha power may reflect mind-wandering when perceptual demands are reduced, e.g., during a monotonous driving situation (Lin et al., 2016). This assumption is further supported by the observation, that the effect of an increase in Alpha power with time on task cannot be observed when task demands are high (Fairclough and Venables, 2006), because participants remain involved in a given task when it is challenging.

Ongoing frontal midline Theta power was found to increase with increasing task demands (Jensen and Tesche, 2002; Onton et al., 2005). It could be shown that a high pre-stimulus Theta power is related to a successful encoding in memory task (Scholz et al., 2017). As frontal Theta activity has been associated with various aspects of executive functioning, it has been proposed as a general marker for cognitive control (Cavanagh and Frank, 2014; Cavanagh and Shackman, 2015). Theta power also increases with time on task (Wascher et al., 2014). As this increase even occurs when the task is demanding (Fairclough and Venables, 2006), it is thus likely that ongoing theta reflects the effort to keep performance high (Wascher et al., 2014; Arnau et al., 2017).

These measures have been also applied to investigate pro- and reactive driving behavior, however, not in distinct scenarios, but rather to characterize different driving styles. In two studies it has been shown that reactive driving behavior goes along with increased steering variability and enhanced alpha power at posterior sites (Garcia et al., 2017; Karthaus et al., 2018).

In order to test pro- and reactive driving and its interaction with time on task (and thereby possible mental fatigue), we created two driving scenarios in which either strategy was predominantly required for good performance. Participants either drove along a straight road and experienced displacements of their car by varying crosswind (re-active), while another group of participants drove along a winded road (pro-active). EEG was measured while driving, and the oscillatory power in general and specifically in Alpha and Theta frequency bands over frontal and posterior brain areas were analyzed. It was expected that the ability to pro-actively control the driving path leads to increased task engagement and consequently to a decrease of Alpha activity. Theta activity should not vary across tasks as long as task demands are comparable.

## Materials and Methods

### Participants

A total of 30 healthy participants took part in the experiment, with 14 of them performing the re-active (7 female, mean age 25.1 years) and 16 the pro-active (8 female, mean age 24.1 years) driving task. All participants were active car drivers, driving at least twice a week during the last three years. They received 10 € per hour for participation in the experiment. None of the participants reported any neurological or psychiatric disorder. All reported normal hearing and normal or corrected to normal vision. They provided informed written consent prior to entering the experiment. The study was approved by the local ethics committee of the Leibniz Research Centre for Working Environment and Human Factors.

### Task and Procedure

Both tasks were set up in a static driving simulator (ST Sim; ST Software B.V. Groningen, Netherlands).

In the re-active driving condition, participants drove on a straight two-lane road. Lane keeping was distracted by a systematically varying sinusoid lateral force resulting from different road slopes. This force simulated periods of crosswind that continuously shifted the vehicle to the left and right, according to a complex signal of eight different superimposed and phase-delayed sine waves (1/25.6, 1/17, 1/12.8, 1/10.2, 1/8.6, 1/7.2, 1/6.4 and 1/5.6 Hz). In the pro-active task, participants drove on a one-lane road with curves of varying radii. Both scenarios were set up in monotonous grassland without any additional visual distraction. Driving speed was held constant at 31 mph. Participants were instructed to keep the vehicle on the track (respectively driving lane in the re-active condition) as accurately as possible.

The strength of the crosswind and the radii of the curves varied across segments (duration 2 min each) randomly between three task load levels (low, middle, high), in which the amplitude of crosswind (respectively radii) were modulated. The high task load condition was adjusted in pilot experiments to amplitudes that allowed participants to keep the car on the track for about 95% of the time. The low task load condition was in both tasks to go along a straight road without distraction. In the middle task load condition the amplitude of the cross wind and the curve radii were exactly in the middle between these two conditions.

Before each task load segment started, a short transfer-interval (duration 1 s) was introduced to smooth the transfer between adjacent segments and avoid abrupt changes. Three different task load segments in randomized order were combined to triplets. The first triplet (6 min overall) served as practice period, in which the participants became familiar with the experimental task. This triplet was followed by nine experimental triplets that were separated into three blocks (with three triplets each) for data analyses. Thus, the experimental block lasted for 54 min, without any break or interruption. While driving a sequence of short tones was presented continuously (sound level 70 dB(A), duration 100 ms, stimulus-onset asynchrony 1000 ms) that should be ignored by the participants. Tone stimuli were initially invented in this series of experiments in order to investigate the Mismatch Negativity (MMN; e.g., Näätänen et al., 2007) as an indicator of mental resources. MMN turned out to be not reliably modulated by experimental manipulations in the present experiment, so we skipped it for the sake of clarity. Given their uniform and periodic character, it appears unlikely that these stimuli systematically changed the oscillatory response or lead to differences in oscillatory response between the experimental conditions.

### Data Recording

For EEG recording 64 scalp electrodes placed according to the International 10–10 system and a “BioSemi active 2” system (BioSemi, Netherlands, USA) were used (sampling rate 2048 Hz, bandwidth DC—140 Hz, electrode impedance below 10 kΩ). Six additional electrodes positioned around both eyes were used for electrooculography to measure horizontal and vertical eye positions. Two additional electrodes were placed on the left and right mastoids.

### Data Analysis

For the processing of the behavioral and EEG data, MATLAB 2016b (The MathWorks Inc., Natick, MA, USA) with the open source toolbox EEGLab (Delorme and Makeig, 2004) was used. Statistical analyses were done using RStudio 1.0.136 (RStudio, Inc., Boston, MA, USA) with R 3.3.2 (R Core Team, 2016). The figures have been created using Veusz^1^.

#### Behavioral Data

Time off track, as the main index for individual accuracy, was defined as the percentage of time, the car left the right driving lane (re-active driving) or the track (pro-active driving). Steering variability, indicating workload (Verwey and Veltman, 1996), was defined as mean number of turns of the steering wheel within one second segments. Finally, steering velocity, served as an indicator for motor activity. It was extracted from the first derivate of the recording of steering position. It should be noted that these parameters diverge from those reported in previous studies in which only crosswind was investigated (e.g., Wascher et al., 2016). These modifications are based on the fact that driving lane variability cannot be applied to turn driving, because pro-active strategies like cutting the corner would lead to an erroneous reduction of performance measures. Time off track appeared to be the only measure of behavioral performance that was applicable to both tasks.

All parameters were entered into a mixed-design ANOVA with the between-subject factor TASK (2; re-active, pro-active) and the within-subject factors TASK LOAD (3; low, medium, high) and TIME ON TASK (3; Blocks 1, 2, 3).

#### EEG Data

**Figure 1:**
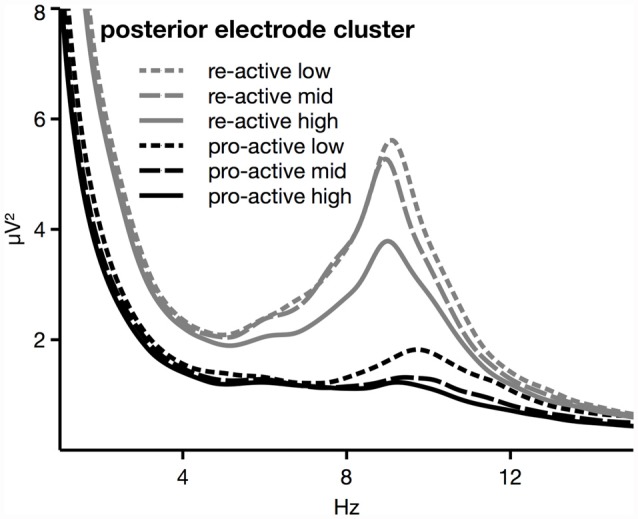
Spectral distribution as measured at posterior electrodes. An overall shift towards higher power is visible in re-active (gray lines) compared to pro-active (black lines) driving. The different lines within tasks reflect different levels of task load.

Broken channels were detected based on kurtosis and probability criteria in data bandpass filtered from 0.1 Hz to 35 Hz and excluded from further analysis. Before performing an Independent Component Analysis (ICA), the data was down-sampled to 256 Hz and band-pass filtered again from 1 Hz to 30 Hz in order to obtain more stable Independent Component (IC) solutions. The data was then segmented into 1 s long epochs around the onset of the tones. Artifacted segments were then detected and rejected automatically. From the remaining segments, a random sample comprising 30 min of data were drawn and used as basis for the ICA. The resulting ICs were than written back into the 2048 Hz sampled data. ICs representing artifacts were detected and rejected using the ADJUST algorithm (Mognon et al., 2011). Again, artifacted segments were rejected automatically (rejection rate 21.3% on average, number of segments for analyses between 1876 and 2941). Finally, previously rejected channels were interpolated. For the analyses of frequency spectra, FFTs were calculated on the extracted segments. Because of a substantial shift (higher power across a wide range of frequencies in the re-active task) that was visible in the raw spectra when pro- and re-active driving were compared (see Figure 1), a two-step analysis was chosen. Firstly, to address the different levels in general power, total power between 1 Hz and 30 Hz was calculated. Thereafter, the mean power was extracted for the Theta (4–7.5 Hz) and the Alpha band (8–12 Hz) and both were normalized on the total power as measured at the same electrodes. The range for each frequency band was selected based on visual inspection of the overall spectrograms (see also Figure 1). In order to minimize spectral overlaps, the width of the window for theta activity was slightly reduced compared to a preceding study (Wascher et al., 2016). Relative power (percentage of total power) was entered into analyses. All analyses were performed separately on the average values of two electrode quadruples defined along the midline (anterior: F1, Fz, F2, FCz; posterior: PO3, POz, Pz, PO4). The electrode groups were selected to cover the main areas where Alpha and Theta effects of mental fatigue and task load have been reported so far (see Wascher et al., 2014). Additionally, analyses of absolute power are given for better comparison to previous studies. Total power, percentages of total power in the Theta and Alpha band and absolute power values were entered into mixed-design ANOVAs with the between-subject factor TASK (2; re-active, pro-active) and the within-subject factors TASK LOAD (3; low, medium, high), and TIME ON TASK (3; Blocks 1, 2, 3).

## Results

### Behavioral Data

**Figure 2:**
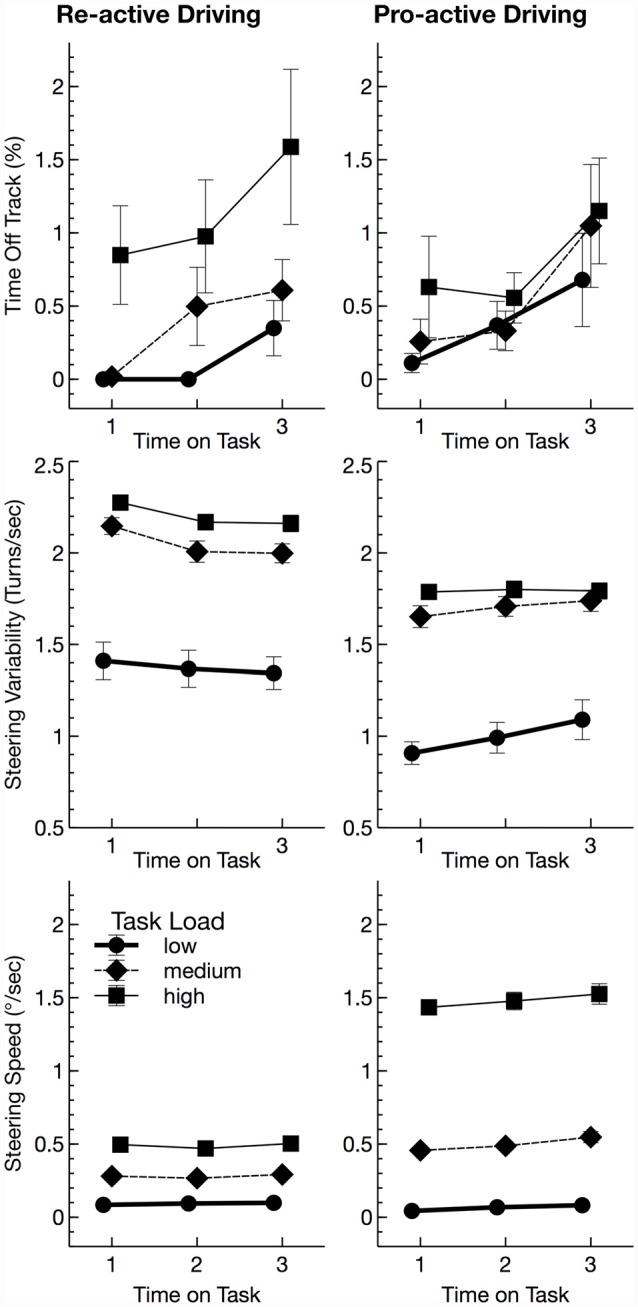
Behavioral parameters (mean values and standard errors of means). Tasks were adjusted for demands based on the percentage of time off track. This led to increased steering variability in the re-active task for the subjects needed to correct their line more often and to increased steering speed in the pro-active task.

Time off track was comparable across TASKs, *F*_(1,28)_ = 0.01, *p* = 0.913, $\eta_{\text{p}}^{2}$ < 0.01 (Figure 2). It increased with TASK LOAD, *F*_(2,56)_ = 9.31, *p* = 0.003, $\eta_{\text{p}}^{2}$ = 0.25, and with TIME ON TASK, *F*_(2,56)_ = 7.44, *p* = 0.005, $\eta_{\text{p}}^{2}$ = 0.21. None of the interactions reached significance.

Steering variability was larger for re-active driving, *F*_(1,28)_ = 26.04, *p* < 0.001, $\eta_{\text{p}}^{2}$ = 0.48, and with increasing TASK LOAD, *F*_(2,56)_ = 243.91, *p* < 0.001, $\eta_{\text{p}}^{2}$ = 0.90 (Figure 2). Additionally, an interaction of task by TIME ON TASK was found, *F*_(2,56)_ = 12.03, *p* < 0.001, $\eta_{\text{p}}^{2}$ = 0.30, that was due a decrease of steering variability with TIME ON TASK for re-active driving, *F*_(2,28)_ = 10.01, *p* = 0.001, $\eta_{\text{p}}^{2}$ = 0.42, but rather an increase for pro-active driving later in the experiment, *F*_(2,28)_ = 3.87, *p* = 0.053, $\eta_{\text{p}}^{2}$ = 0.22.

Steering velocity, on the other hand was larger for pro-active driving, *F*_(1,28)_ = 152.42, *p* < 0.001, $\eta_{\text{p}}^{2}$ = 0.84 (Figure 2). It increased with TASK LOAD, *F*_(2,56)_ = 1374.24, *p* < 0.001, $\eta_{\text{p}}^{2}$ = 0.98, and with TIME ON TASK, *F*_(2,56)_ = 7.94, *p* = 0.004, $\eta_{\text{p}}^{2}$ = 0.22. Additionally, steering velocity showed interactions of TASK by TASK LOAD, *F*_(2,56)_ = 456.59, *p* < 0.001, $\eta_{\text{p}}^{2}$ = 0.94, and TASK by TIME ON TASK. In both task, steering velocity increased with TASK LOAD (re-active: *F*_(2,28)_ = 714.02, *p* < 0.001, $\eta_{\text{p}}^{2}$ = 0.98; pro-active: *F*_(2,28)_ = 935.26, *p* < 0.001, $\eta_{\text{p}}^{2}$ = 0.99) which was inherent in the task design. The increase of steering velocity with TIME ON TASK, was less pronounced for re-active driving, *F*_(2,28)_ = 2.93, *p* = 0.070, $\eta_{\text{p}}^{2}$ = 0.17, compared to pro-active driving, *F*_(2,28)_ = 6.72, *p* = 0.013, $\eta_{\text{p}}^{2}$ = 0.32.

### EEG Data

**Figure 3:**
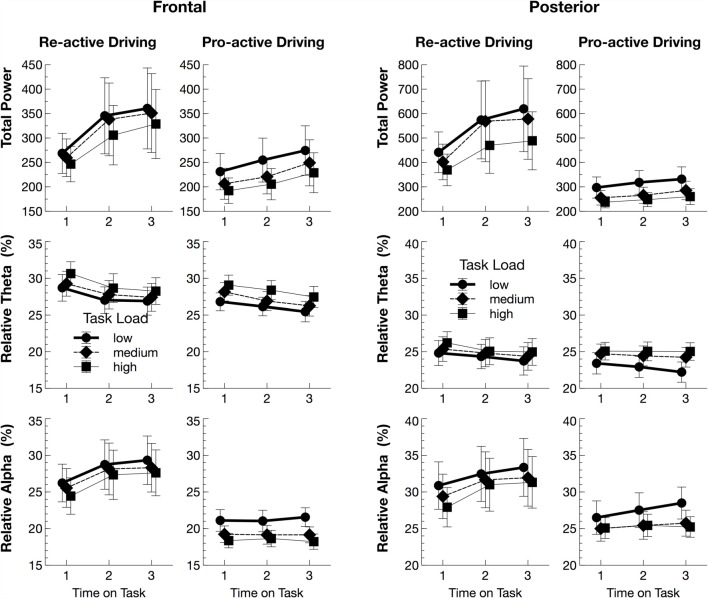
Mean values (with standard errors of mean) for total power and relative power in the Alpha and Theta band for frontal and posterior electrodes.

Total power decreased with increasing TASK LOAD for both frontal (*F*_(2,56)_ = 16.64, *p* < 0.001, $\eta_{\text{p}}^{2}$ = 0.37) and posterior (*F*_(2,56)_ = 10.46, *p* = 0.001, $\eta_{\text{p}}^{2}$ = 0.27) sides (Figure 3). The increase with TIME ON TASK was significant for frontal (*F*_(2,56)_ = 6.16, *p* = 0.009, $\eta_{\text{p}}^{2}$ = 0.18), but only marginal for posterior (*F*_(2,56)_ = 3.39, *p* = 0.053, $\eta_{\text{p}}^{2}$ = 0.11) sides. Moreover, there was a marginal effect of TASK on posterior total power (*F*_(1,28)_ = 3.12, *p* = 0.088, $\eta_{\text{p}}^{2}$ = 0.10), being larger in the re-active, than pro-active, driving task.

The analysis of the relative power values (Figure 3) indicated that the relative Theta power was lower with lower TASK LOAD (frontal: *F*_(2,56)_ = 35.05, *p* < 0.001, $\eta_{\text{p}}^{2}$ = 0.56; posterior: *F*_(2,56)_ = 23.34, *p* < 0.001, $\eta_{\text{p}}^{2}$ = 0.45), and decreased with TIME ON TASK (frontal: *F*_(2,56)_ = 13.43, *p* < 0.001, $\eta_{\text{p}}^{2}$ = 0.32; posterior: *F*_(2,56)_ = 3.64, *p* = 0.045, $\eta_{\text{p}}^{2}$ = 0.12). Moreover, for the posterior electrode side, there was a slight interaction of TASK by TASK LOAD, *F*_(2,56)_ = 3.19, *p* = 0.067, $\eta_{\text{p}}^{2}$ = 0.10, suggesting that the decrease of relative Theta power with decreasing task load was more pronounced in the pro-active, than re-active, driving task.

Absolute Theta power increased with lower TASK LOAD (frontal: *F*_(2,56)_ = 5.43, *p* = 0.022, $\eta_{\text{p}}^{2}$ = 0.16; posterior: load *F*_(2,56)_ = 5.11, *p* = 0.026, $\eta_{\text{p}}^{2}$ = 0.15) and tended to do so at least frontally with TIME ON TASK (frontal: *F*_(2,56)_ = 2.92, *p* = 0.091, $\eta_{\text{p}}^{2}$ = 0.09; posterior: *F*_(2,56)_ = 1.94, *p* = 0.168, $\eta_{\text{p}}^{2}$ = 0.06). Posterior Theta additionally tended to be increased in the re-active task (*F*_(1,28)_ = 3.57, *p* = 0.069, $\eta_{\text{p}}^{2}$ = 0.11).

The relative Alpha power demonstrated an opposite pattern. It decreased with increasing TASK LOAD (frontal: *F*_(2,56)_ = 33.24, *p* < 0.001, $\eta_{\text{p}}^{2}$ = 0.54; posterior: *F*_(2,56)_ = 11.05, *p* = 0.001, $\eta_{\text{p}}^{2}$ = 0.28), and increased with TIME ON TASK (frontal: *F*_(2,56)_ = 4.86, *p* = 0.020, $\eta_{\text{p}}^{2}$ = 0.15; posterior: *F*_(2,56)_ = 6.40, *p* = 0.009, $\eta_{\text{p}}^{2}$ = 0.19). Moreover, the frontal relative Alpha power was higher in the re-active task compared to the pro-active task, *F*_(1,28)_ = 5.60, *p* = 0.025, $\eta_{\text{p}}^{2}$ = 0.17. The difference in Alpha power even increased, according to an interaction of TASK by TIME ON TASK, *F*_(2,56)_ = 4.47, *p* = 0.026, $\eta_{\text{p}}^{2}$ = 0.14. Also, there was a slight interaction of TASK by TASK LOAD, *F*_(2,56)_ = 3.38, *p* = 0.059, $\eta_{\text{p}}^{2}$ = 0.11, indicating that the decrease of frontal relative Alpha power with increasing TASK LOAD was more pronounced in the pro-active, than re-active, driving task. No effects of TASK were found for relative Alpha power for the posterior site, all *p* > 0.10, $\eta_{\text{p}}^{2}$ < 0.08.

Absolute Alpha power increased with decreasing TASK LOAD (frontal: *F*_(2,56)_ = 12.65, *p* < 0.001, $\eta_{\text{p}}^{2}$ = 0.31; posterior: *F*_(2,56)_ = 6.14, *p* = 0.015, $\eta_{\text{p}}^{2}$ = 0.18), and with TIME ON TASK (frontal: *F*_(2,56)_ = 3.61, *p* = 0.054, $\eta_{\text{p}}^{2}$ = 0.11, posterior: *F*_(2,56)_ = 2.59, *p* = 0.105, $\eta_{\text{p}}^{2}$ = 0.08).

To sum up, there were quite general effects on the total power of oscillatory activity (which increased with time on task and decreased with increasing task load). While absolute power in both frequency bands showed more or less the same pattern, specific effects were found for the relative power in Alpha and Theta bands. In particular, an effect of the task performed was observed, with the frontal relative Alpha power being lower for pro-active compared to re-active driving.

## Discussion

The main aim of the present study was to uncover the role of intentional control for enduring monotonous driving tasks by means of psychophysiological parameters reflecting a drivers’ mental states.

EEG parameters, in particular oscillatory activity, have a long tradition in user state examination. It was reported repeatedly that the spectrum of the EEG slows down with long term task performance, in other words that low frequency bands such as Alpha or Theta increase in power. Assuming a close relationship between power in low frequency bands and mental fatigue, Lal and Craig (2001) even proposed that EEG measure may serve as measure for detecting a driver’s fatigue, hence preventing accidents. However, the main problem with this approach is that all these measures vary not only with mental fatigue, but also depend on other cognitive parameters such as task load (Wascher et al., 2016), working memory demands, or attention (for a review see Klimesch, 2012). Intentional control is an additional candidate to affect task engagement and therefore to interact with mental fatigue.

In the present study, participants either had to go along a straight road with varying crosswind (re-active) or to drive a winding road (pro-active). The two tasks were adjusted with respect to difficulty in a way that comparable performance was on average achieved in both tasks. To obtain this goal, stronger turns had to be driven in the pro-active task, because they were predictable and therefore easier to be handled. Consequently, and as intended, no significant differences in time off track were observed. There were, however, differences in steering variability, which is assumed to be an index of task load (Verwey and Veltman, 1996; Ahlstrom et al., 2012), suggesting that task load might be higher in re-active driving, despite the adjustments of task demands. Most of this effect can be assigned to correction behavior that should be widely reduced when steering behavior can be anticipated. Interestingly, steering variability increased with time on task in pro-active driving and decreased in re-active driving. Both effects went along with decreasing driving performance and indicate that participants reduced engagement into the task. Re-active corrections were reduced and potentially reduced pro-active planning of the driving task led to an increased need to correct the steering angle. Steering velocity, on the other hand, was larger in pro-active driving, reflecting higher motor activity in cornering, especially when taking narrow bends.

The effects of task load, time on task and task condition were mirrored in oscillatory EEG parameters. First of all, we replicated our previous findings of increasing Alpha and Theta power with decreasing task load and time on task (see Wascher et al., 2016). The same pattern, however, was obtained for total power, indicating this to be a global effect on lower frequency bands, which has been previously reported to be related to mental fatigue (see e.g., Lal and Craig, 2001). The strong modulation with task load, however, indicates that it might be also related to boredom and attentional withdrawal that the driver experienced when the driving situation is less demanding and long-lasting. In addition, driving along a straight road with crosswind compensation might have been more boring (and consequently more prone to attentional withdrawal) than turn taking on a winding road, probably related to a slightly larger global power in the re-active, than pro-active, task condition. However, effects in total power may as well be due to a bias across the two samples tested here. It is noteworthy that all other effects found for total power accord to results reported for the lower frequency bands with mental fatigue and task load. Thus, high variability in this measure is not likely the cause of this potential bias.

Due to the fact that the task effect in total power may have camouflaged specific effects in the selected frequency bands, we focused on the relative power in Alpha and Theta bands. Support for this approach comes from the observation that absolute power in both investigated frequency bands varied very similarly to this rather general measure.

The relative Alpha activity corresponds to the total EEG power and absolute Alpha power, being increased with lower task load and increasing time on task for both frontal and posterior areas. In addition, the relative frontal Alpha power showed differences in task conditions, and was larger in the re-active, than pro-active, scenario. Higher Alpha activity has been related to a mental state of attentional disengagement (Baldwin et al., 2017). Thus, the task effect on this measure suggests that re-active behavior of crosswind compensation may have temped the drivers to withdraw attentional resources, while taking bends may have resulted in a more focused driving activity. The interaction of task and time on task indicated an even increasing relative frontal Alpha power in the re-active task, while relative Alpha power remained at a constant low level throughout the pro-active driving task. Also this finding would be in accordance of Alpha activity in terms of attentional engagement.

In contrast to relative Alpha activity, relative Theta did not follow this overall pattern. It increased with high task load, which might reflect an increase in the need for cognitive control in the more demanding driving situations (see also Cavanagh and Frank, 2014). In the same sense, the decrease in relative Theta power with time on task could be due to learning, resulting in a decreasing amount of cognitive resources needed to manage these driving situations. Most interestingly, Theta power appeared to drop in the pro-active task with low task load. In this condition actually, participants went along a straight road without anticipating any distraction. These results apparently contradict previous studies that reported increasing Theta power with time on task (Lal and Craig, 2000, 2002; Craig et al., 2012; Wascher et al., 2014, 2016). However, it should be noted that those studies reported absolute power in the Theta band that might be strongly influenced by total power which constantly increases also in the data presented here. This might also explain why the scalp topography of fatigue effects remain fairly stable across frequency bands (see Craig et al., 2012) and does not depict specific effects. In contrast to absolute power, relative power emphasizes effects that go along with changes in the dominance of frequency bands across the spectrogram (Klimesch, 1999). Relative power was employed here on a first sight to overcome the task effect in the comparisons, however, turned out to probable emphasize distinct modulation within single frequency bands. On the other hand, relative power has to be interpreted with caution. Large changes in one frequency band (here Alpha), can cause relative power to decrease in other frequency bands (Theta). This might have modulated effects on relative theta but cannot account for the entire data pattern.

In summary, the present study shows that EEG activity in the low frequency bands is not only modulated by time on task or cognitive demands, but also by the controllability of a task. Both the total power of the EEG and the relative power in the Alpha band increase with time on task, the ease of a route section and low controllability of the driving situation (i.e., in the re-active task). All these results are in accordance with attentional withdrawal as a central risk factor in monotonous tasks. Relative Theta power is the only measure reported here which pattern did not follow this general effects. Both, the increase with higher task load and the decrease with time on task are in accordance with an interpretation of Theta activity in terms of cognitive demands. This argument can be strengthened by the fact that relative theta shows particularly low values in the pro-active sections with low task load. This is the only condition in which it is predictable that no action is needed.

## Author Contributions

EW and SG wrote the manuscript. EW, MK and SG were responsible for the design of the study. SA and IG were responsible for data analysis.

## Conflict of Interest Statement

IG is the owner of “BlindSight GmbH”, a company designing and distributing biosignal acquisition equipment, which was not used in the present study. Thus, the present study is not related to the business activities of this company. The other authors declare that the research was conducted in the absence of any commercial or financial relationships that could be construed as a potential conflict of interest.

## Footnotes

^1^https://veusz.github.io/
